# Dr. John A. Cleveland

**Published:** 1853-01

**Authors:** 


OBITUARY.—*Integer vitæ, scelerisque purus.*—Died, of yellow fever, in Charleston, South Carolina, on the 28th of September, 1852, Dr. JOHN A. CLEVELAND.

It has never been our duty to record the death of a more useful, more estimable and more excellent man than he was; one, who better illustrated that idea of human excellence, which gentleness, truth and charity combine to form. The epitaphs of most men claim as much for their subjects as truth will permit, when they record strict integrity, general correctness of intention, and impulsive goodness; to say no more of him, were to leave much unsaid, were to leave a space for a foible or a fault for which he substituted an amiability or an excellence. The duties of life with him were not alone the greetings of the market-place, or the exact fulfilment of those obligations which convenience or expediency enjoins. He sought those opportunities of adding to the sum of human happiness, which others often are content should seek them. He never waited, but went to do good. His benefactions oftener anticipated than followed the embarrassment or the mortification of being obliged to ask. This trait of his character has repeatedly impressed itself in our intercourse with him; and his memory will ever be associated with the unsolicited bestowal of many a kindness.

If this appears to be the encomium of his too partial friend, we are willing to hear the hardest censure which his enemy, if he has left one, dares to pronounce. We do not claim that he had no frailties; but we do not believe, that any one of his survivors has so few to lament, that he would be willing to take the first stone.

"To live in hearts we leave behind,
Is, not to die."

May his spirit repose with Him who rewards the just. C. D. B.

Dr. Cleveland was the eldest son of Dr. John Cleveland, of Canterbury, Conn., would have been fifty-six years old in January, 1853. He settled in Georgia in 1819—and resided at various times in the counties of Chatham, Burke, Wilkes and Richmond, till the year 1839, when he moved to Charleston, S. C., and settled in the drug business, continuing to practice dentistry till his illness.

Dr. C. commenced the practice of dentistry in the year 1828 or '9, and traveled through many of the middle counties of Georgia, and settled in Augusta in 1832, at which time he married Miss Hariet A. Aldridge, of Herkimer, N. Y.

In 1838, the health of his wife being precarious, by advice he went to St. Thomas, W. I., where his wife died in the spring of 1839. Very few practitioners of dentistry have ever attained the high stand he did among his professional brethren and patrons.

The early medical instruction of his father, who destined him for the profession of medicine, and his extraordinary mechanical genius, gave him greater advantage on the practice of mechanical dentistry, in which branch he had no superior. The instruments with which he commenced and practiced for many years, were of his own manufacture. Indeed, many instruments in his cabinet were his own invention, and several of his professional brethren are indebted for facilities in their practice to his suggestions.

In 1850, he perfected his "Air Chamber Suction Plate," and has since inserted many full sets of teeth upon that principle, which have stood every test, and have given entire satisfaction.

Dr. Cleveland was twice elected and served as vice-president of the American Society of Dental Surgeons, and was held in high estimation by every Fellow of the Association who had the pleasure of knowing him.

